# Synthesis of Azanucleosides through Regioselective Ring-Opening of Epoxides Catalyzed by Sulphated Zirconia under Microwave and Solvent-Free Conditions

**DOI:** 10.3390/molecules17033359

**Published:** 2012-03-15

**Authors:** Celia Xochitl Hernández-Reyes, Deyanira Angeles-Beltrán, Leticia Lomas-Romero, Eduardo González-Zamora, Rubén Gaviño, Jorge Cárdenas, José Antonio Morales-Serna, Guillermo E. Negrón-Silva

**Affiliations:** 1 Departamento de Ciencias Básicas, Universidad Autónoma Metropolitana, Av. San Pablo No. 180, México D.F., C.P. 02200, Mexico; Email: floronix@yahoo.com.mx (C.X.H.-R.); dab@correo.azc.uam.mx (D.A.-B.); 2 Departamento de Química, Universidad Autónoma Metropolitana, Av. San Rafael Atlixco No. 186, México D.F., C.P. 09340, Mexico; Email: llr@xanum.uam.mx (L.L.-R.); egz@xanum.uam.mx(E.G.-Z.); 3 Instituto de Química, Universidad Nacional Autónoma de México, Circuito Exterior, Ciudad Universitaria, México D.F. 04510, Mexico; Email: rgavino@unam.mx (R.G.); rjcp@unam.mx(J.C.); morser@unam.mx (J.A.M.-S.)

**Keywords:** azanucleosides, sulphated zirconia, nucleophilic reaction, regioselective reaction, epoxide’s ring-opening, microwave

## Abstract

New azanucleosides were obtained using sulphated zirconia (ZS) as catalyst in the nucleophilic oxirane ring opening reaction of 1-allyl-3-(oxiran-2-ylmethyl)pyrimidine-2,4(1*H*,3*H*)-dione and 1-allyl-5-methyl-3-(oxiran-2-ylmethyl)-pyrimidine-2,4(1*H*,3*H*)-dione, with (*S*)-prolinol. The new templates were obtained with good yields following a route which exploits the reactivity of epoxides in the presence of sulphated zirconia as catalyst. The key step was carried out using microwave and solvent-free conditions and proceeds with high selectivity.

## 1. Introduction

The use of solid materials as reusable catalysts in chemical synthesis contributes significantly to simplify diverse processes like separation, purification and isolation of the reaction products, to optimize the conversion and performance, as well as to decrease pollutants generation. In addition, as the reaction can be carried out in solvent-less conditions, the process turns out to be even cleaner and simpler [1]. The catalytic activity of the sulphated zirconia relates to a combination of acid Brønsted and Lewis sites, although also to other factors such as: (a) surface defects; (b) presence of paramagnetic species like Zr^+3^; and (c) suitable crystallization and phase concentration periods activate the tetragonal phase [2]. Sulphated zirconia is a catalyst, that can be easily recovered and reused; therefore, it is not surprising that a large variety of organic reactions can be catalyzed by sulphated zirconia [3,4].

In this context and as part of our ongoing research program, we have been interested since eight years ago in the preparation of sulphated zirconia and its use in organic reactions. We have proved that sulphated zirconia is an excellent catalyst in such reactions as: cyclization of 1,4-dicarbonyl compounds and substituted anilines to tetrahydroindolones [5], chemoselective synthesis of acylals from aromatic aldehydes and their deprotections [6,7], synthesis of 3,4-dihydropyrimidin-2(1*H*)-ones using multicomponent reactions [8], synthesis of amino alcohols by ring-opening of epoxides [9,10] and nucleophilic addition to carbonyl compounds [11]. Presently, we desire to extend this scope to the synthesis of azanucleosides which are biologically interesting molecules, because of their activity against human tumor cell lines [12,13] and for the treatment of patients with myelodysplactic syndromes [14].

**Figure 1:**
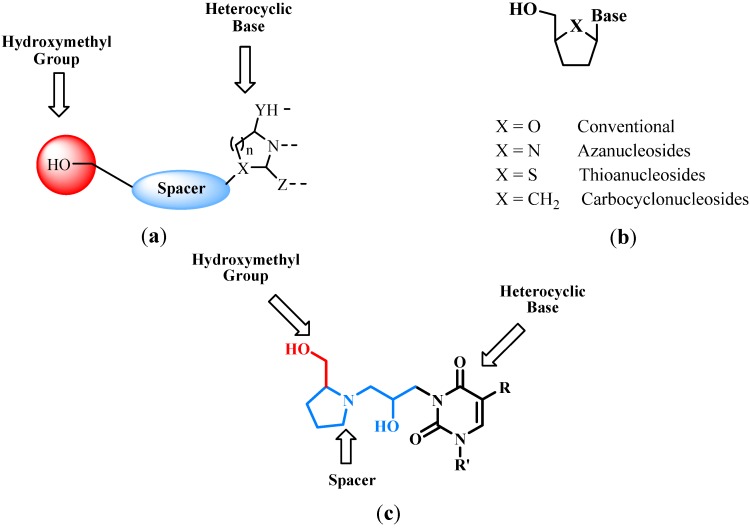
(**a**) Basic structure of nucleosides; (**b**) Type of nucleosides; (**c**) New kind of nucleosides’ analogues.

The modification of nucleosides has been recognized as an important research area to improve their antiviral activities [15,16,17,18,19,20,21,22,23,24,25,26]. Each biologically active nucleoside is constituted by the heterocyclic base moiety, a furanose ring and a hydroxymethyl group (Figure 1a), which participate in the recognition process to achieve biological activity via the phosphorylation of hydroxymethyl group [27].

The synthesis of new active nucleoside analogues has been focused in the search of nucleoside derivatives with major antiviral activities that has been demonstrated by commercial antivirals, but with lower toxicity. Among them, azanucleosides are one of the most interesting modifications reported in the literature [28]. Structurally, azanucleosides are analogues of nucleosides with ribofuranosyl moieties, where the oxygen is replaced by a nitrogen atom (Figure 1b), which allows inhibiting the hydrolysis of the glycosidic bond by glycohydrolases [29,30,31,32]. With this background, we considered the possibility to insert a pyrrolidine ring and hydroxymethyl group in the position *N*-3 of pyrimidinedione aiming to generate a new kind of nucleosides analogues (Figure 1c). Thus, in the present paper we describe a new protocol of the azanucleosides synthesis by nucleophilic opening of epoxides using sulphated zirconia as catalyst.

## 2. Results and Discussion

### 2.1. Sulphated Zirconia Characterization.

**Figure 2:**
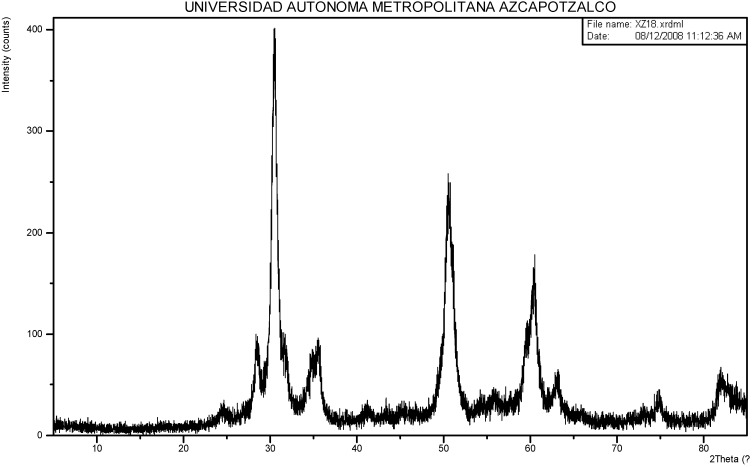
Sulphated zirconia diffractogram.

**Figure 3:**
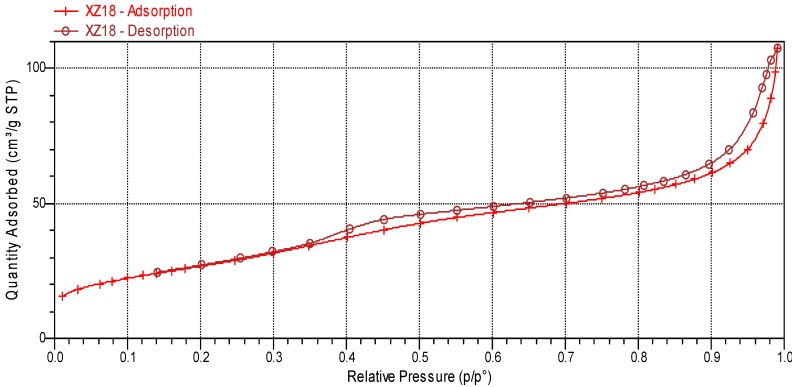
Sulphated zirconia nitrogen adsorption-desorption isotherm.

Sulphated zirconia shows a crystalline structure that is preponderantly tetragonal, as proved through the XRD peaks shown in Figure 2. The nitrogen adsorption-desorption isotherm (Figure 3) corresponds to type IV of the IUPAC classification; the hysteresis loop indicates a uniform pore size distribution. The BET area is 93 m^2^·g^−1^, while the average pore size was 50.69 Å and pore volume 0.12 cm^3^·g^−1^. Ample discussion about the characterization of this material has been provided in our previous works [5,6,7,8,9,10,11].

### 2.2. Azanucleosides Synthesis

**Scheme 1:**
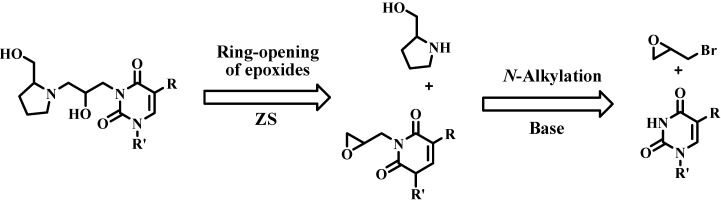
Corrected linguistically retrosynthetic analysis.

The choice of starting material was dictated by the type of reaction that we planned to employ to bond the principal fragments of the nucleoside: heterocyclic base moiety, spacer and hydroxymethyl group. Our strategy for the synthesis of azanucleosides is outlined in Scheme 1.

**Scheme 2:**
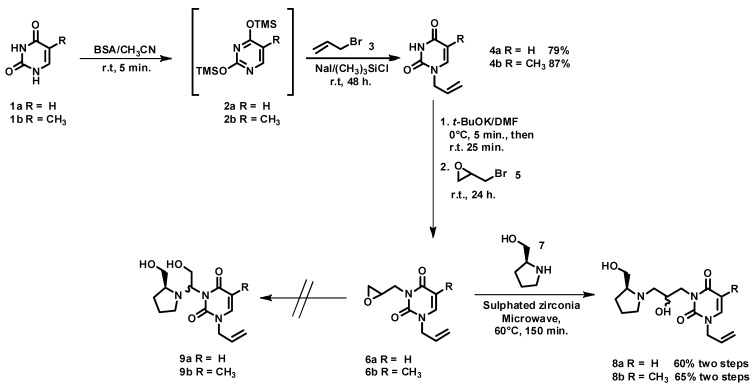
Synthesis of azanucleosides.

Based on a previously described methodology [33] and our experience in the alkylation reactions of nucleosides [34], we decided to obtain compounds **4a** and **4b** via *N*-alkylation of the base in position *N*-1. Thus, uracil (**1a**) and thymine (**1b**) were transformed into *N*-1-allyluracil (**4a)** and *N*-1-allylthymine (**4b**) via the sequential addition procedure of *N*,*O*-bis(trimethylsilyl) acetamide (BSA) as silylating agent to give the intermediates **2a** and **2b**, and then the alkylation reaction using allyl bromide (**3**) in the presence of NaI and thrimethylsilyl chloride (TMSCl) afforded **4a** and **4b**. The reaction procedure is shown in Scheme 2. The presence of allyl group in the position *N*-1 can be explained by our interest in the future preparation of nucleoside libraries by modification in that position.

Next, the desired products **8a** and **8b** were synthesized by another sequential procedure, where **4a** and **4b** were alkylated first in position *N*-3 to give the epoxides **6a** and **6b**. The high instability of those compounds impeded purification, and they were used crude in the next step. Thus, in a second step, the catalytic activity of the sulphated zirconia was evaluated in the nucleophilic ring-opening of epoxides **6a** and **6b** with (*S*)-prolinol (**7**), under microwave irradiation and solvent-free conditions. To our delight, the azanucleosides **8a** and **8b** were obtained as diastereoisomeric mixtures in 60 and 65% yield, respectively (Scheme 2). In both cases, the diastereoisomers showed a similar R_f_ in TLC (EtOAc-MeOH-TEA, 90:9:1), and for this reason it was impossible to separate the mixtures on a chromatographic column using silica gel as stationary phase. The ^1^H-NMR spectra showed the presence of a multiplet at δ 4.1, which was assigned to the methylene protons contiguous to position *N*-3. The signal at δ 4.1 integrates for three protons, with the third proton corresponding to the methine proton of the tertiary alcohol. The ^1^H-^1^H COSY and HMBC spectra supported that connectivity. However, the identification of each diastereoisomer was not possible, due to the complexity of the spectra. Finally, it is noteworthy that in both cases the formation of other isomers (**9a** and **9b**) was not observed, which confirms the high selectivity of the ring-opening reaction. These results agree with those previously described by our group [10], where the use of microwave and solvent-free conditions led to decreasing reaction times without modification of regioselectivity.

**Scheme 3:**
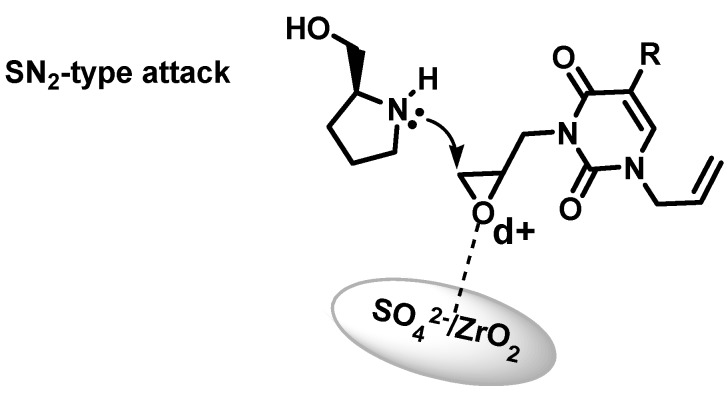
Proposed reaction mechanism for the regioselective ring-opening of epoxides.

The selectivity observed in the present work is consistent with an SN_2_-type attack of the prolinol nitrogen at the less hindered carbon of the epoxide (Scheme 3). Similar behavior has been described by Reddy and co-workers in alkyl indoles [35] and Das and co-workers in an imidazole and styrene oxide system [36]. Additionally, we had observed that our catalyst can be easily recovered and reused for at least three cycles without any significant decreases in yield and regioselectivity [7,9].

## 3. Experimental

### 3.1. General

All the chemicals were purchased from Aldrich Chemical Co., and used without further purification, unless stated otherwise. Yields refer to chromatographically and spectroscopically (^1^H and ^13^C) homogeneous materials. All glassware utilized was flame-dried before use. Reactions were monitored by TLC carried out on 0.25-mm Macherey Nagel silica gel plates. Developed TLC plates were visualized under a short-wave UV lamp and by heating plates that were dipped in Ce(SO_4_)_3_. Flash column chromatography (FCC) was performed using flash silica gel (230–400) and employed solvent of a polarity which correlates with TLC mobility. Microwave reactions were performed in a CEM Labmate^®^ microwave reactor. NMR experiments were conducted on a Bruker-Avance 500 MHz, instrument using CDCl_3_ (99.9% D) as solvent, with chemical shifts (δ) referenced to internal standards CDCl_3_ (7.26 ppm ^1^H, 77.0 ppm ^13^C) or Me_4_Si as an internal reference (0.00 ppm). Chemical shifts are in parts per million (ppm).

The X-ray diffraction (XRD) studies were performed in a Philips X’Pert instrument using Cu-Kα radiation (45 kV, 40 mA). The nitrogen adsorption-desorption isotherms were obtained in a Micromeritics ASAP 2020 equipment at −196 °C. The reaction products were analyzed by GC-FID (Agilent Technologies 6890N) equipped with a column HP-5 with the following program; 70–150 °C (25 °C/min) for 1.00 min, then 150–210 °C (10 °C/min) for 2 min and finally to 210–280 °C (10 °C /min) for 3 min, injector to 250 °C, detector to 280 °C. The mass spectra was obtained by GC-MS (Agilent Technologies 6890N, Detector 5973), in the chemical ionization mode using methane as ionization gas.

### 3.2. Catalyst Synthesis [5]

Zirconium *n*-propoxide (20 mL, 70% n-propanol) was mixed with 2-propanol (30 mL) and stirred with a magnetic bar. Then, distilled water (3.2 mL) was mixed with sulfuric acid (1 mL, 98%); this solution was added dropwise in order to carry out the hydrolysis and gelation of the zirconium *n*-propoxide. The solid was dried at 80 °C until complete alcohol evaporation and then calcined in air at 600 °C for 6 h.

### 3.3. Synthesis of Azanucleosides

*N-1-Allyluracil *(**4a**) [33]. Under a nitrogen atmosphere, uracil (**1a**, 3.2 g, 28.58 mmol) was placed with distilled acetonitrile (43 mL) in a ball flask provided with stopper and magnetic stirrer. The mixture was stirred at room temperature for 5 minutes. Then, *N*,*O*-bis(trimethylsilyl) acetamide (BSA, 17.64 mL, 72.14 mmol) was added, the mixture was kept under agitation until it became transparent. Then, allyl bromide (**3**, 5 mL, 57.77 mmol), sodium iodide (NaI, 4.8 g, 32.02 mmol) and trimethylsilyl chloride (TMSCl, 3.6 mL, 28.46 mmol) were added successively, and the reaction mixture was kept under vigorous agitation at room temperature for 48 h. When the reaction finished, ethyl acetate (20 mL) was added and it was neutralized with a 10% wt solution of sodium bicarbonate (NaHCO3, 70 mL). The crude product was filtered and the liquid phase was extracted using ethyl acetate (80 mL). The organic phase was evaporated under reduced pressure. The crude was purified by chromatography in a silica column using 40:60 ether/ethyl acetate as eluent. *N*-1-allyluracil (**4a**) was obtained as a white solid in 79% yield. m.p.: 105–106 °C (lit. [37] m.p.: 103–105 °C); GC-MS for C_7_H_8_N_2_O_2_ (m.w.: 152.09 g/mol): [M+1]^+^ = 153, [M+29]^+^ = 181, and [M+41]^+^ = 193; IR (cm^−1^): 3,114, 3,009, 2,926, 2,813, 1,667, 1,641, 1,463, 1,408, 1,338, 1,242, 1,200, 920, 833, 632; ^1^H-NMR (CDCl_3_): δ 10.07 (1H, brs), 7.19 (1H, d, *J* = 7.9 Hz), 5.88 (1H, ddt, *J* = 17.1, 10.3, 5.9 Hz), 5.75 (1H, d, *J* = 7.9 Hz), 5.33–5.25 (2H, m), 4.37 (2H, dt, *J* = 5.9, 1.5 Hz). ^13^C-NMR (CDCl_3_): δ 164.0, 150.9, 143.7, 131.4, 119.3, 102.4, 49.9 [38].

*N-1-Allylthymine* (**4b**) [33]. Under a nitrogen atmosphere, thymine (**2b**, 3.60 g, 28.58 mmol) was placed with distilled acetonitrile (43 mL) in a ball flask provided with stopper and magnetic stirrer. The mixture was stirred at room temperature during 5 minutes. Then, BSA (17.64 mL, 72.14 mmol) was added, and the mixture was kept under agitation until it became transparent. Then, allyl bromide (3, 5 mL, 57.77 mmol), NaI (4.8 g, 32.02 mmol) and TMSCl (3.6 mL, 28.46 mmol) were added successively, and the reaction mixture was kept under vigorous agitation at room temperature for 48 h. When the reaction finished, ethyl acetate (20 mL) was added and the reaction mixture was neutralized with a 10% wt solution of NaHCO_3_ (55 mL). The crude product was filtered and the liquid phase was extracted using ethyl acetate (80 mL). The organic phase was evaporated under reduced pressure. The crude was purified by chromatography in a silica column using 40:60 ether/ethyl acetate as eluent. *N*-1 allylthymine (**4b**) was obtained as a white solid in 87% yield. m.p.: 108–110 °C, (lit. [38] m.p.: 112 °C); GC-MS for C_8_H_10_N_2_O_2_ (m.w.: 166.20 g/mol): [M+1]^+^ = 167, [M+29]^+^ = 195 and [M+41]^+^ = 207; IR (cm^−1^): 3,147, 3,029, 2,829, 1,683, 1,459, 1,424, 1,344, 1,217, 1,141, 908, 893, 869, 714, 610; ^1^H-NMR (CDCl_3_): δ 9.60 (1H, brs), 6.98 (1H, q, *J* = 1.2 Hz), 5.87 (1H, ddt, *J* = 17.1, 10.3, 5.8 Hz), 5.32–5.24 (2H, m), 4.34 (2H, dt, *J* = 5.8, 1.5 Hz), 1.93 (3H, d, *J* = 1.3 Hz); ^13^C-NMR (CDCl_3_): δ 164.3, 150.9, 139.6, 131.7, 119.1, 110.9, 49.7, 12.2 [38].

*1-Allyl-3-(2-hydroxy-3-((S)-2-(hydroxymethyl)pyrrolidin-1-yl)propyl)pyrimidine-2,4(1H,3H)-dione) *(**8a**). *N*-1-Allyluracil (**4a**, 0.304 g, 2 mmol) in DMF (5 mL) were placed in a round-bottom flask. Then, potassium *tert*-butoxide (*t*-BuOK, 0.494 g, 4.4 mmol) was added at 0 °C under a nitrogen atmosphere over 5 min. Then, the mixture was stirred for 25 min at room temperature. Finally, epibromohydrin (**5**, 0.36 mL, 4.4 mmol) was added and the reaction mixture was stirred for 24 h at room temperature. After this time, the mixture was diluted with methylene chloride (5 mL) and the product was recovered by extractions with methylene chloride (5 × 15 mL) and water (5 mL) and the organic extract was dried over Na_2_SO_4_ and concentrated under vacuum to give **6a**. The crude product was used directly in the next step. In a closed reaction system there were placed **6a** (0.398 g, as crude product) and (*S*)-prolinol (**7**, 0.18 mL, 1.81 mmol) with sulphated zirconia (100 mg). The reaction mixture was irradiated in a microwave reactor (CEM Labmate^®^) under vigorous agitation at 60 °C for 150 min (initial power 50 W). After the reaction time methanol (10 mL) was added, the catalyst was recovered by filtration and washed with acetone (20 mL). The organic product was dried under reduced pressure. The residue was purified by flash column chromatography on silica gel (EtOAc/MeOH, 95:5) to give **8a** as a colorless oil. ^1^H-NMR (CDCl_3_): δ 7.26 (1H, d, *J* = 7.8 Hz), 5.89 (1H, ddt, *J* = 17.0, 10.1, 5.7 Hz), 5.79 (1H, d, *J* = 7.8 Hz), 5.30 (2H, m), 4.39 (2H, d, *J* = 5.7 Hz), 4.10 (3H, m), 3.62 (1H, dd, *J* = 11.4, 3.6 Hz), 3.42 (1H, dd, *J* = 11.4, 7.2 Hz), 3.24 (1H, m), 2.85 (1H, dd, *J* = 12.6, 9.3 Hz), 2.76 (1H, m), 2.53 (1H, dd, *J* = 13.12, 2.7 Hz), 2.38 (1H, q, *J* = 8.4 Hz), 1.8 (4H, m); ^13^C-NMR (CDCl_3_): δ 164.1, 152.0, 142.7, 131.4, 119.4, 101.7, 68.1, 67.5, 66.2, 63.1, 62.8, 59.8, 59.5, 56.0, 54.9, 51.3, 45.3, 27.1, 23.5; HRMS (FAB) calc. for C_15_H_23_N_3_O_4_ 309.1689, found 309.1654.

*1-Allyl-3-(2-hydroxy-3-((S)-2-(hydroxymethyl)pyrrolidin-1-yl)propyl)-5-methylpyrimidine-2,4(1H,3H)-dione * (**8b**)*.* *N*-1 Allylthymine (**4b**, 0.332 g, 2 mmol) in DMF (5 mL) was placed in a round-bottom flask. Then, *t*-BuOK (0.494 g, 4.4 mmol) was added at 0 °C under a nitrogen atmosphere over 5 min. Then, the mixture was stirred for 25 min at room temperature. Finally, epibromohydrin (**5**, 0.36 mL, 4.4 mmol) was added and the reaction mixture was stirred for 24 h at room temperature. After this reaction time, the mixture was diluted with methylene chloride (5 mL) and the product was recovered by extractions with methylene chloride (5 × 15 mL) and water (5 mL) and the organic extract was dried over Na_2_SO_4_ and concentrated under vacuum to give **6a**. The crude product was used directly in the next step. In a closed reaction system were placed **6b** (0.454 g as crude product) and (*S*)-prolinol (**7**, 0.19 mL, 1.94 mmol) with sulphated zirconia (100 mg). The reaction mixture was irradiated in a microwave reactor (CEM Labmate^®^) under vigorous agitation at 60 °C during 150 min (initial power 50 W). Next methanol (10 mL) was added, the catalyst was recovered by filtration and washed with acetone (20 mL). The organic product was dried under reduced pressure. The residue was purified by flash column chromatography on silica gel (EtOAc/MeOH, 95:5) to give **8b** as a colorless oil. ^1^H-NMR (CDCl_3_): δ 7.02 (1H, brs), 5.79 (1H, ddt, *J* = 17.0, 10.1, 5.7 Hz), 5.25 (2H, m), 4.29 (2H, d, *J* = 5.7 Hz), 4.10 (3H, m), 3.61 (1H, dd, *J* = 11.4, 3.6 Hz), 3.52 (1H, dd, *J* = 11.4, 7.2 Hz), 3.27 (1H, m), 2.93 (1H, m), 2.61 (1H, dd, *J* = 12.9, 2.1 Hz), 2.70 (1H, m), 2.53 (1H, q, *J* = 8.4 Hz), 1.80 (4H, m), 1.85 (3H, brs); ^13^C-NMR (CDCl_3_): δ 164.8, 152.0, 138.98, 131.8, 119.2, 110.2, 67.4, 66.9, 62.2, 59.9, 55.0, 51.2, 45.4, 26.9, 24.4, 12.9; HRMS (FAB) calc. for C_16_H_25_N_3_O_4_ 323.1845, found 323.1839.

## 4. Conclusions

We have demonstrated that sulphated zirconia is an efficient catalyst in the regioselective nucleophilic opening of the oxiranes 1-allyl-3-(oxiran-2ylmethyl) pyrimidine-2,4-(1*H*,3*H*)-dione (**6a**) and 1-allyl-5-methyl-3-(oxiran-2-ylmethyl) pyrimidine-2,4-(1*H*,3*H*)-dione (**6b**) with (*S*)-prolinol to give the azanucleosides **8a** and **8b** with good yield. The advantage of our approach is that the epoxide ring-opening was carried out under solvent-free conditions and with short reaction time using microwave irradiation. We believe that our protocol will find use in the efficient synthesis of a new kind of nucleosides analogues and facilitate the synthesis of novel bioactive molecules.
